# HeuDiConv — flexible DICOM conversion into structured directory layouts

**DOI:** 10.21105/joss.05839

**Published:** 2024-07-03

**Authors:** Yaroslav O. Halchenko, Mathias Goncalves, Satrajit Ghosh, Pablo Velasco, Matteo Visconti di Oleggio Castello, Taylor Salo, John T. Wodder, Michael Hanke, Patrick Sadil, Krzysztof Jacek Gorgolewski, Horea-Ioan Ioanas, Chris Rorden, Timothy J. Hendrickson, Michael Dayan, Sean Dae Houlihan, James Kent, Ted Strauss, John Lee, Isaac To, Christopher J. Markiewicz, Darren Lukas, Ellyn R. Butler, Todd Thompson, Maite Termenon, David V. Smith, Austin Macdonald, David N. Kennedy

**Affiliations:** 1 Center for Open Neuroscience, Department of Psychological and Brain Sciences, Dartmouth College, Hanover, NH, USA; 2 Department of Psychology, Stanford University, CA, USA; 3 McGovern Institute, Massachusetts Institute of Technology, Cambridge, MA, USA; 4 Flywheel Exchange LLC, Minneapolis, MN, USA; 5 University of California, Berkeley, Berkeley, CA, USA; 6 Perelman School of Medicine, University of Pennsylvania, Philadelphia, PA, USA; 7 Institute of Neuroscience and Medicine, Brain & Behaviour (INM-7), Research Center Jülich, Jülich, Germany; 8 Institute of Systems Neuroscience, Medical Faculty, Heinrich Heine University Düsseldorf, Düsseldorf, Germany; 9 Department of Biostatistics, Johns Hopkins Bloomberg School of Public Health, Baltimore, MD, USA; 10 Emeritus of Department of Psychology, Stanford University, CA, USA; 11 Department of Psychology, University of South Carolina, Columbia, SC, USA; 12 Masonic Institute for the Developing Brain, University of Minnesota, Minneapolis, MN, USA; 13 Minnesota Supercomputing Institute, University of Minnesota, Minneapolis, MN, USA; 14 Human Neuroscience Platform, Fondation Campus Biotech Geneva, Geneva, Switzerland; 15 Department of Brain and Cognitive Sciences, Massachusetts Institute of Technology, Cambridge, MA, USA; 16 Department of Psychology, University of Texas at Austin, Austin, TX, USA; 17 McConnell Brain Imaging Centre, McGill University, Montreal, QC, Canada; 18 Data Science and Sharing Team, National Institute of Mental Health, Bethesda, MD, USA; 19 Institute for Glycomics, Griffith University, QLD, Australia; 20 Department of Psychology, Northwestern University, Evanston, IL, USA; 21 Biomedical Engineering Department, Faculty of Engineering, Mondragon University, Mondragon, Spain; 22 BCBL, Basque center on Cognition, Brain and Language, San Sebastian, Spain; 23 Department of Psychology and Neuroscience, Temple University, Philadelphia, PA, USA; 24 Departments of Psychiatry and Radiology, University of Massachusetts Chan Medical School, Worcester, MA, USA

## Summary

In order to support efficient processing, data must be formatted according to standards that are prevalent in the field and widely supported among actively developed analysis tools. The Brain Imaging Data Structure (BIDS) (Gorgolewski et al., 2016) is an open standard designed for computational accessibility, operator legibility, and a wide and easily extendable scope of modalities — and is consequently used by numerous analysis and processing tools as the preferred input format in many fields of neuroscience. HeuDiConv (Heuristic DICOM Converter) enables flexible and efficient conversion of spatially reconstructed neuroimaging data from the DICOM format (quasi-ubiquitous in biomedical image acquisition systems, particularly in clinical settings) to BIDS, as well as other file layouts. HeuDiConv provides a multi-stage operator input workflow (discovery, manual tuning, conversion) where a manual tuning step is optional and the entire conversion can thus be seamlessly integrated into a data processing pipeline. HeuDiConv is written in Python, and supports the DICOM specification for input parsing, and the BIDS specification for output construction. The support for these standards is extensive, and HeuDiConv can handle complex organization scenarios that arise for specific data types (e.g., multi-echo sequences, or single-band reference volumes). In addition to generating valid BIDS outputs, additional support is offered for custom output layouts. This is obtained via a set of built-in fully functional or example heuristics expressed as simple Python functions. Those heuristics could be taken as a template or as a base for developing custom heuristics, thus providing full flexibility and maintaining user accessibility. HeuDiConv further integrates with DataLad (Halchenko et al., 2021), and can automatically prepare hierarchies of DataLad datasets with optional obfuscation of sensitive data and metadata, including obfuscating patient visit timestamps in the git version control system. As a result, given its extensibility, large modality support, and integration with advanced data management technologies, HeuDiConv has become a mainstay in numerous neuroimaging workflows, and constitutes a powerful and highly adaptable tool of potential interest to large swathes of the neuroimaging community.

## Statement of need

Neuroimaging is an empirical research area which relies heavily on efficient data acquisition, harmonization, and processing. Neuroimaging data sourced from medical imaging equipment, and in particular magnetic resonance imaging (MRI) scanners, can be exported in numerous formats, among which DICOM (Digital Imaging and Communications in Medicine) is most prominent. DICOM data are often transmitted to PACS (Picture Archiving and Communication Systems) servers for archiving or further processing. Unlike in clinical settings, where data are interfaced with directly from PACS in the DICOM format, in neuroimaging research, tools typically require data files in the NIfTI (NIfTI Data Format Working Group, 2003--) format which directly stores images as 3D or 4D objects and restricts metadata to the most useful attributes. Tools such as dcm2niix (Li et al., 2016) can be used to convert DICOM files into NIfTI files, and can extract metadata fields not covered by the NIfTI header into sidecar .json files. However, the scope of such tools is limited, as they do not extend to organizing multiple NIfTI files for different subjects and scanning sessions within a study.

HeuDiConv was created in 2014 to provide flexible tooling so that labs may rapidly and consistently convert collections of DICOM files into collections of NIfTI files in customizable file system hierarchies. As manual file renaming and metadata reorganization is tedious and error prone, automation is preferable, and this is a consistent focus of HeuDiConv.

Since the inception of HeuDiConv in 2014, the BIDS standard (Gorgolewski et al., 2016) was established. The BIDS standard formalizes data file hierarchies and metadata storage in a fashion which, due to its community-driven nature, is both highly optimized and widely understood by analysis tools. Since then, DICOM conversion to NIfTI files contained within a BIDS hierarchy has emerged as the most frequent use-case for HeuDiConv.

## State of the field

**Figure 3:**
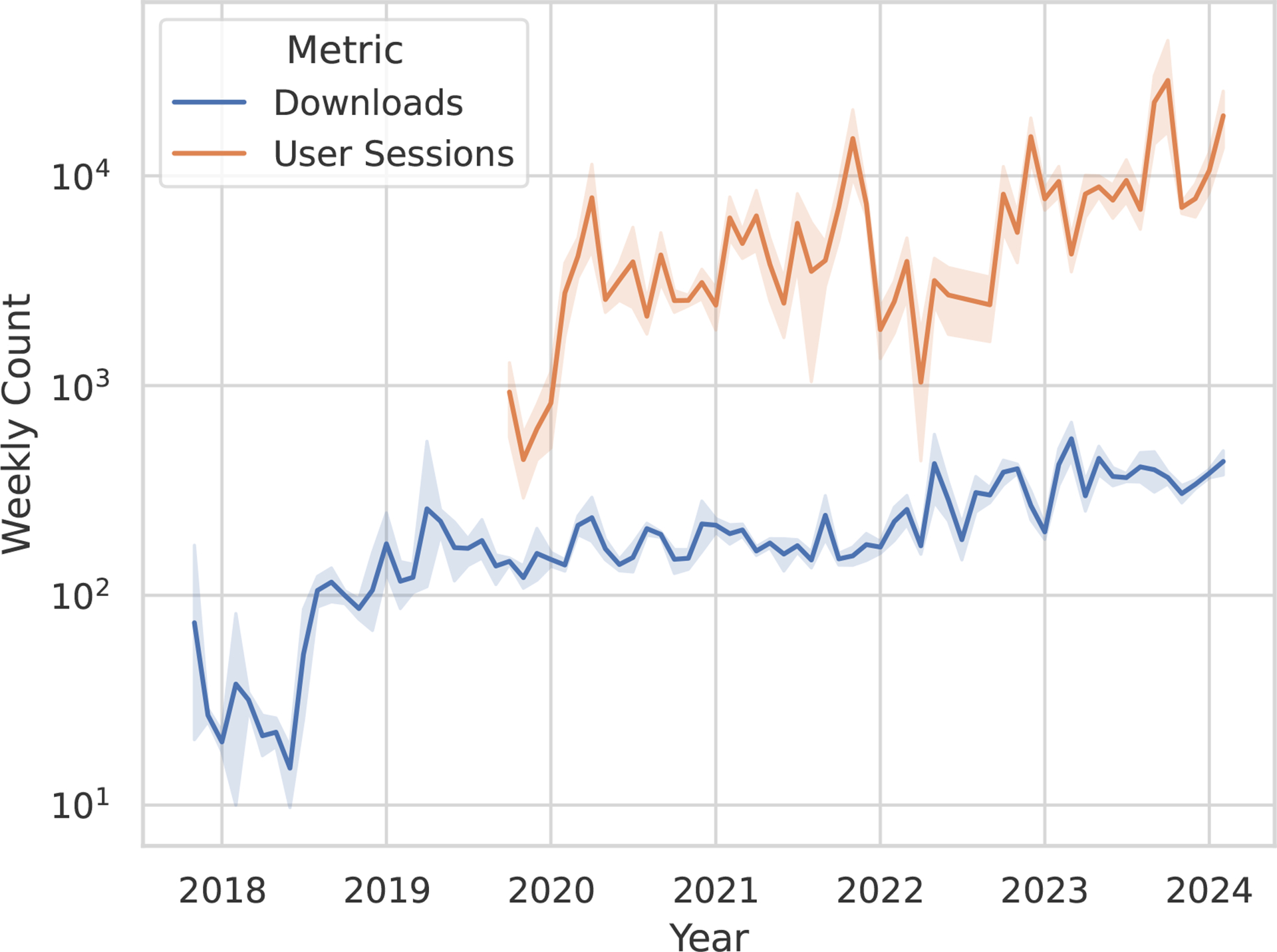
Downloads experienced an initial sharp rise after the ReproNim HeuDiconv training event at OHBM in mid 2018, and have subsequently followed a positive trend along with the usage — exceeding 1000 sessions per week — in the data collection interval. Depicted are weekly download and confirmed session estimates, averaged per month, with a 95% confidence interval. User session estimates for July and August 2022 are linearly extrapolated from the nearest neighbour. Download counts are sourced from PyPI, the Python community repository; whereas user session counts are sourced from Etelemetry, an infrastructure for verifiable research impact, which end-users can disable to protect privacy.

Conversion of data to BIDS is acknowledged to remain one of the challenges (Poldrack et al., 2024) which lead to the proliferation of converters to BIDS (see https://bids.neuroimaging.io/benefits#converters). As BIDS grows and evolves, so do the requirements for conversion tools. New converters are being developed, and existing ones are being updated to accommodate new data types and modalities. HeuDiConv is one of the most widely used converters, and its usage statistics keep growing (see Figure 3. It is actively maintained and developed to keep up with the latest BIDS standards and community needs. Although it could be used without any knowledge of Python programming, it is designed to be extensible and customizable, and to allow for the development of custom heuristics to handle specific data types and modalities. Unlike GUI- or Web-UI based tools, such as EZ-BIDS (Levitas et al., 2024), which might be easier for novices to use, HeuDiConv is designed to be used in a command-line environment, and is thus well-suited for integration into automated data processing pipelines.

## Overview of HeuDiConv functionality

HeuDiConv has been developed to implement logic commonly used across labs (grouping DICOMs, extracting metadata, converting individual sequences, populating standard BIDS files, etc.). It allowed individual groups to customize **how** files should be organized and named while driving custom decisions through the conventions and desires of those individual groups. Such decision making is implemented in *HeuDiConv heuristics*, which are implemented as Python modules following some minimalistic specified interfaces documented in HeuDiConv documentation (https://heudiconv.readthedocs.io/en/latest/heuristics.html). HeuDiConv, if instructed to operate in BIDS mode (--bids flag) with a heuristic providing base naming instructions, helps to organize the files in the hierarchy defined by the BIDS standard. It also ensures files are named according to the BIDS specifications, including complex composite recordings such as those associated with multi-echo sequences.

### Exemplar heuristics

#### Convertall

The convertall heuristic is the simplest heuristic which expresses no knowledge or assumptions about anything and can be used as a template to develop new heuristics or to establish initial mapping for manual naming of the sequences in the “manual curation” step.

#### StudyForrest phase 2

The studyforrest_phase2 heuristic is a small sample heuristic developed for the StudyForrest (Hanke et al., 2014) project, and demonstrates custom conversion into BIDS datasets.

#### ReproIn

The ReproIn heuristic was initially developed at the Dartmouth Brain Imaging Center (DBIC) to automate data conversion into BIDS for any neuroimaging study performed using the center’s facilities. The core principle behind ReproIn is the reduction of operator interaction required to obtain BIDS datasets for acquired data. It is achieved by ensuring that reference MRI sequences on the instrumentation are organized and named in a consistent and flexible way, such that upon usage in any experimental protocol they will encode the information required for fully automatic conversion and repositing of the resulting data.

In case of correct specification and absent operator errors, such as mis-typed subject or session IDs, it can be fully automated, and work is ongoing to make such deployments turnkey. Visit the ReproIn project page http://reproin.repronim.org to discover more.

To try it out, one can download sample reproin_dicom.zip and use the following command:

heudiconv --files reproin_dicom.zip -f reproin --bids -o bids_datasets

to produce a BIDS dataset in the bids_datasets/output/Patterson/Coben/ sub-directory. This demonstrates the ability to automate conversion using ReproIn for all studies in the center. The Tutorials section in the HeuDiConv documentation provides more examples across for different scenarios.

## Adoption and usage

As a citeable resource RRID:SCR_017427, Heudiconv has already 6 mentions in papers at time of writing. There is a growing number of downloads from PyPI and uses of HeuDiConv (see Figure 3). Over 40 BIDS datasets were converted over to BIDS with HeuDiConv at Dartmouth Brain Imaging Center (DBIC), using the ReproIn heuristic developed there. HeuDiConv was found to be used for PET data conversion (Jamadar et al., 2021), shared as OpenNeuro ds003382 (Sharna Jamadar et al., 2020). Moreover, the HeuDiConv approach inspired the development of fw-heudiconv (FlywheelTools: Software for HeuDiConv-Style BIDS Curation On Flywheel) (Tapera et al., 2021).

## External dependencies

HeuDiConv uses specialized tools and libraries:

- datalad (Halchenko et al., 2021) (RRID: SCR_003931) enables managing produced datasets as version controlled repositories.
- dcm2niix (Li et al., 2016) is used for the conversion from DICOM to NIfTI and initial versions of sidecar .json files,
- etelemetry and filelock are used as supplementary utilities,
- neurodocker (Kaczmarzyk et al., 2023) (RRID:SCR_017426) is used to produce Dockerfile from which docker images are built,
- nipype (Gorgolewski et al., 2011) (RRID:SCR_002502) to interface dcm2niix and extra metadata invocations,
- pydicom (Mason et al., 2022) (RRID:SCR_002573) and dcmstack for DICOM analysis and extraction of extra metadata to place to BIDS sidecar files,
- pytest formalizes unit and integration testing.

## Figures and Tables

**Figure 1:**
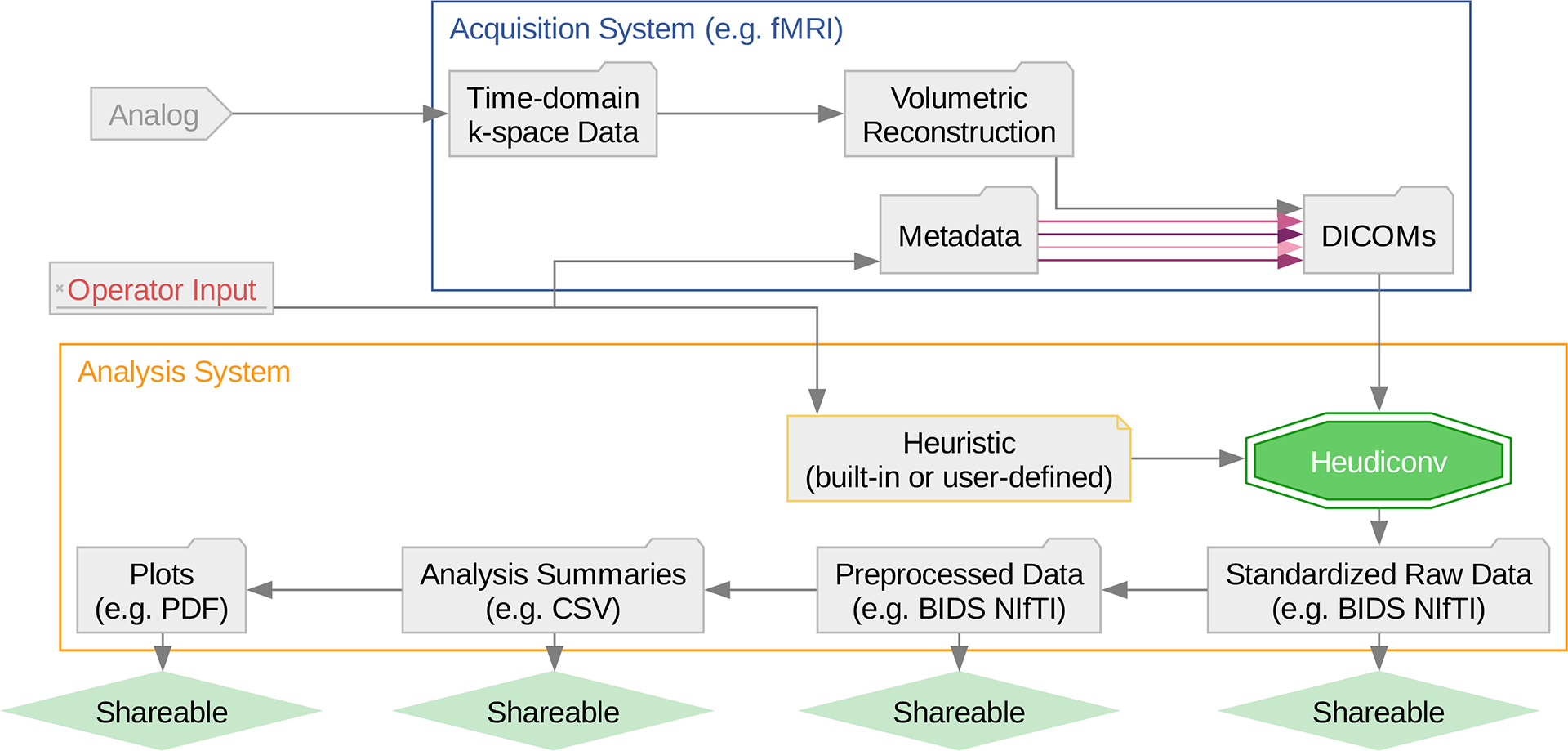
HeuDiConv automates the keystone conversion step in reproducible data handling, without compromising operator flexibility. The showcased set-up depicts a 2-machine infrastructure, with heudiconv operating on the same machine as subsequent analysis steps for data in a standardized and shareable representation. For more advanced usage at institutions with dedicated infrastructure, HeuDiConv can operate on an additional third machine, which then interfaces between the depicted two machines and is dedicated to data repositing, versioning, and backup.

**Figure 2:**
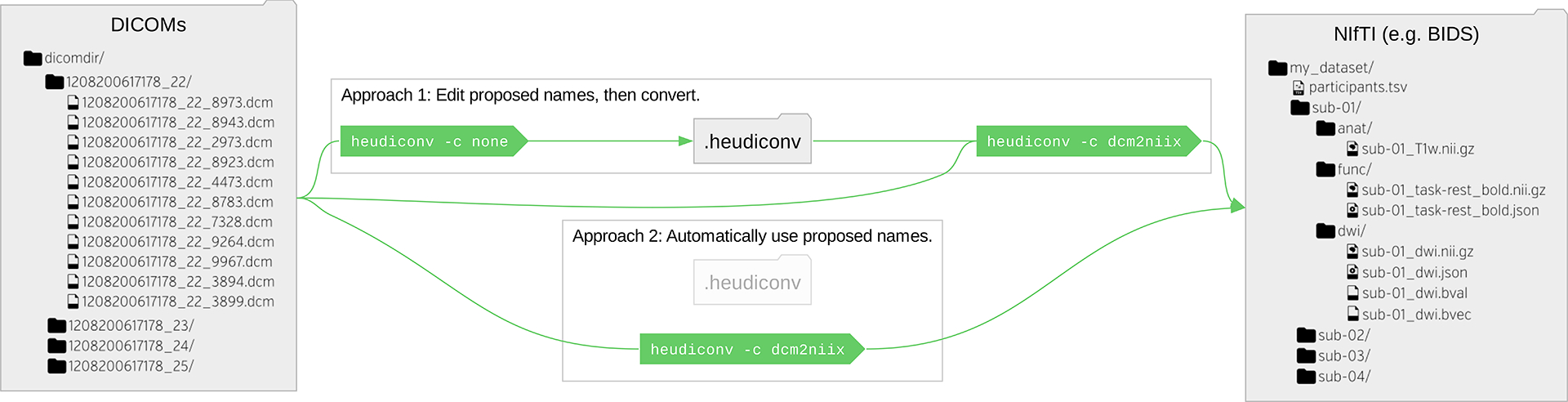
HeuDiConv conversion and layout is controlled via heuristics (custom or provided built-ins) either with manual tune up of proposed filenames or fully automated. The heudiconv application can be used with the -c none parameter to generate by heuristic a list of filenames for the user to edit, before invoking the conversion to be performed via the -c dcm2niix option to use the dcm2niix tool. The process is idempotent, and specifying the -c dcm2niix option can automatically convert without seeking user tune up of proposed filenames.
